# A Target Coverage Scheduling Scheme Based on Genetic Algorithms in Directional Sensor Networks

**DOI:** 10.3390/s110201888

**Published:** 2011-02-01

**Authors:** Joon-Min Gil, Youn-Hee Han

**Affiliations:** 1 School of Computer and Information Communications Engineering, Catholic University of Daegu, 330 Geumnak-Ri, Hayang-Eup, Gyeongsan-Si, Gyeongbuk 712-702, Korea; E-Mail: jmgil@cu.ac.kr; 2 Advanced Technology Research Center, Korea University of Technology and Education, 1800 ChungJeollo, Byeongcheon-Myeon, Cheonan-Si, Chungnam 330-708, Korea

**Keywords:** target coverage, directional sensors, network lifetime, greedy algorithms, genetic algorithms

## Abstract

As a promising tool for monitoring the physical world, directional sensor networks (DSNs) consisting of a large number of directional sensors are attracting increasing attention. As directional sensors in DSNs have limited battery power and restricted angles of sensing range, maximizing the network lifetime while monitoring all the targets in a given area remains a challenge. A major technique to conserve the energy of directional sensors is to use a node wake-up scheduling protocol by which some sensors remain active to provide sensing services, while the others are inactive to conserve their energy. In this paper, we first address a Maximum Set Covers for DSNs (MSCD) problem, which is known to be NP-complete, and present a greedy algorithm-based target coverage scheduling scheme that can solve this problem by heuristics. This scheme is used as a baseline for comparison. We then propose a target coverage scheduling scheme based on a genetic algorithm that can find the optimal cover sets to extend the network lifetime while monitoring all targets by the evolutionary global search technique. To verify and evaluate these schemes, we conducted simulations and showed that the schemes can contribute to extending the network lifetime. Simulation results indicated that the genetic algorithm-based scheduling scheme had better performance than the greedy algorithm-based scheme in terms of maximizing network lifetime.

## 1. Introduction

Wireless sensor networks (WSNs) have been employed in various fields such as environmental monitoring, battlefield surveillance, smart spaces, *etc*. [1]. WSNs are typically composed of large numbers of sensors that have sensing, data processing, and communication functionalities. In WSNs, *coverage* determines how well an area (or points) of interest is monitored or tracked by sensors [2]. There are three types of coverage based on what is to be covered: area coverage, target (discrete point) coverage, and barrier coverage. In this paper, we focus on target coverage [3,4] in a randomly deployed sensor network where the density of sensor nodes is sufficiently high to monitor all targets.

For the target coverage problem, it is essential that sensors monitor all the targets continuously for as long as possible. Each sensor has a limited battery. Once sensors are randomly scattered, it is hardly possible to replace or recharge their battery [5,6]. When a sensor completely exhausts its battery power, it cannot be used for target coverage anymore. The sensor simply disappear in the WSN and remaining available sensors should continue to execute target coverage. Therefore, the problem of maximizing the network lifetime while covering all the targets is an important issue. To achieve this purpose, each sensor should minimize its battery power consumption in an energy-efficient manner. Typically, sensors have four types of radio state: transmit, receive, idle, and sleep [4]. We can denote transmit, receive, and idle states as active states, because each of these states consumes more energy than the sleep state. Therefore, scheduling schemes to properly alternate between active and sleep states, *i.e.*, node wake-up scheduling protocols [7], are a promising method of maximizing the network lifetime.

Many attempts have been made to maximize network lifetime based on node wake-up scheduling protocols. In particular, these studies have assumed that WSNs have omnidirectional sensors, each of which can sense an omnidirectional range at each instance [4,8]. Recently, directional sensors such as camera/video sensors [9–11], ultrasonic sensors [12], infrared sensors [13], *etc.*, have been developed and networks consisting of such sensors, *i.e.*, *directional sensor networks (DSNs)*, are widely used. Each sensor in a DSN has a sensing range as a sector. Unlike WSNs, target coverage in DSNs is determined by both location and direction of sensors. This feature of DSNs makes target coverage scheduling more complex. Therefore, maximizing the network lifetime of DSNs remains a challenge problem. Nevertheless, few studies have addressed the target coverage problem in DSNs.

In this paper, we discuss the problem of target coverage scheduling in DSNs whose directional sensors have limited battery capacity and are randomly deployed to cover all targets. The connectivity issue of the deployed directional sensors is not considered in solving this problem. Instead, we assume that there are mobile robots to move to each fixed sensor and collect the sensed data [14] or a DSN is connected due to the large communication range of directional sensors. We describe a Maximum Set Covers for DSNs (MSCD) problem, which entails finding the cover sets that monitor all the targets in an energy-efficient way and maximizing the network lifetime by assigning different scheduling times to each cover set. As reported previously [15], this problem is known to be NP-complete. To resolve the problem, we first devise a greedy heuristic algorithm that has the advantage of finding a solution faster than other algorithms. Due to its local search, however, the greedy algorithm may fail to find an optimal solution for target coverage that maximizes the network lifetime of DSNs. As another solution, we also introduce a genetic algorithm, based on evolutionary global search techniques, to find optimal cover sets in DSNs. Simulation results verified that these two schemes can solve the MSCD problem. They also showed that the genetic algorithm-based target scheduling scheme is better capable of finding cover sets with an extended network lifetime as compared with the greedy algorithm-based scheme.

The rest of this paper is organized as follows. Section 2 provides the related work on target coverage scheduling in wireless sensor networks. In Section 3, we formally define the MSCD problem. A target coverage scheduling scheme based on a greedy algorithm to solve the problem is also presented in this section. In Section 4, we propose another target coverage scheduling scheme based on a genetic algorithm. This section also provides detailed descriptions of our genetic algorithm. In Section 5, we present the performance evaluation of these schemes with simulations. Section 6 concludes the paper. This paper is an updated and extended version of [16].

## 2. Related Work

The concept of target coverage is a fundamental measure of the quality of service (QoS) of the sensing function. The goal is to have each target in the physical space of interest within the sensing range of at least one sensor. A survey on target coverage problems in wireless sensor networks is presented in [17,18]. The initial works relevant to our study are [3,4]. [3] introduced the target coverage problem, where disjoint sensor sets are modeled as disjoint cover sets, such that every cover set completely monitors all targets; the problem was called *Maximum Set Covers (MSC)* and proved to be NP-complete in the study. The MSC problem was reduced to a maximum flow problem, which was then modeled as mixed integer programming. This problem was further extended in [4], where sensors were not restricted to participation in only disjoint sets, *i.e.*, a sensor could be active in more than one set. In [4], two heuristic algorithms were proposed to solve the MSC problem: Linear Programming (LP)-MSC and greedy-MSC. The authors showed that the greedy-MSC had lower complexity and running time than the LP-MSC. They also demonstrated that the greedy-MSC increased the network lifetime to a greater extent than the LP-MSC. Even if the MSC problem is NP-complete, most approaches have used heuristic strategies, such as LP and greedy algorithms. Several recent studies have been performed to solve the MSC problem using the optimization capability of genetic algorithms [19,20]. In [19], the maximization of cover sets was modeled to extend the network lifetime of wireless sensor networks and then optimized by genetic algorithms. Furthermore, [20] proposed a coverage control scheme in which a genetic algorithm was used to select the minimum number of sensors in a densely deployed environment while monitoring all targets.

However, above related work discussed only the target coverage problem under the WSNs in which sensors have omnidirectional sensing ranges. Directional sensors differ from omnidirectional sensors in that the coverage region of a sensor is determined by both its location and orientation. Therefore, the target coverage problem aiming at directional sensors will be more complicated than that focusing on omnidirectional sensors. This paper is an extension of the MSC problem addressed in [3,4], for the case when sensor nodes can be directional. The initial work relevant to the coverage issue in DSNs was presented in [15]. The authors formulated a Maximum Coverage with Minimum Sensors (MCMS) problem, in which coverage in terms of the number of targets to be covered is maximized, while the number of sensors to be activated is minimized. In contrast, our study focuses on producing as many cover sets as possible while monitoring all targets, thus maximizing the network lifetime. A genetic algorithm was applied in [21] to solve target coverage in DSNs. Given the assumption that targets to be covered had the prescribed priorities, that study attempted to find a minimum set of directional sensors at any instant. In contrast to that work, our work uses a genetic algorithm to find the maximal number of cover sets without exceeding the available energy of directional sensors, leading to maximal extension of the network lifetime of a DSN.

In summary, our work differs from previous studies in several ways. First, the MSCD problem for target coverage in DSNs is formulated. Second, we also present a heuristic solution with the greedy algorithm for the MSCD problem. Moreover, a global search solution with genetic algorithms is designed to find more cover sets than the heuristic solution. Finally, in the design process of genetic algorithms, we use diverse genetic operations suitable for the MSCD problem, such as a two-dimensional representation method that can encode candidate cover sets into chromosomes, two types of crossover operators (for inter-cover sets and intra-cover sets) that can efficiently search global optimum to solve the MSCD problem, a mutation operator that can tune the orientations of directional sensors in DSNs, and a fitness function that can lead to finding as many cover sets as possible and, at the same time, exhausting the residual energy of directional sensors completely until the network lifetime is extended maximally. There are no reports yet, to the best of our knowledge, about a target coverage scheduling scheme based on genetic algorithms for extending the network lifetime while monitoring all targets in DSNs.

## 3. Maximum Set Covers for DSNs

In this section, we define the Maximum Set Covers for DSNs (MSCD) problem and present a greedy algorithm to solve the problem.

### 3.1. MSCD Problem

Let us consider a DSN composed of *N* sensors, each of which has *W* directions and operates in only one direction with a uniform sensing range at any instant. We also consider that all sensors are randomly scattered to cover *M* targets in a two-dimensional plane. We define *S* = {*s*_1_,*s*_2_,…,*s_N_*} as the set of *N* sensors and *R* = {*r*_1_,*r*_2_,…,*r_M_*} as the set of *M* targets. Unlike a sensor network composed of omnidirectional sensors, a DSN should additionally consider definitions related to sensor directions.

- *D_i,j_*: the *j*th direction of a sensor *s_i_* (*i* = 1,2,…,*N* and *j* = 1,2,…,*W*). We assume that a sensor *s_i_* has no overlap between two neighboring directions.
- *D*: the collection of *D_i,j_* for *i* = 1,2,…,*N* and *j* = 1,2,…,*W* .
- *C_k_* (⊆ *D*): the *k*th set of the directions that cover all targets in *R* such that every element in *C_k_* covers at least one element in *R* and every two elements in *C_k_* cannot belong to the same sensor in *S*. We call this set *C_k_* a *cover set*.
- *R_m_* (⊆ *D*): the set of directions that cover a target *r_m_* for *m* = 1,2,…,*M*.
- *L_i_*: the lifetime of a sensor *s_i_*. We assume that a sensor *s_i_* spends a uniform amount of energy regardless of its direction when it is active.
- *t_k_*: the allocated active time for the *k*th cover set (0 ≤ *t_k_* ≤ 1).

**Figure 1.**
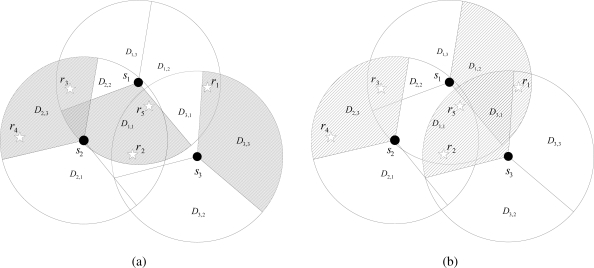
Illustrative example of a directional sensor network. **(a)** cover set {*D*_1,1_, *D*_2,3_, *D*_3,3_}; **(b)** cover set {*D*_1,2_,*D*_2,3_,*D*_3,1_}.

Before formally formulating the target coverage problem in DSNs, we illustrate an example of a DSN in which three directional sensors with three directions can cover five targets. In Figure 1, *r_m_*(1 ≤ *m* ≤ 5) and *s_i_*(1 ≤ *i* ≤ 3) represent a target and a directional sensor, respectively. *D_i,j_*(1 ≤ *i,j* ≤ 3) represents the direction of *s_i_*, and thus *D* = {*D*_1,1_,*D*_1,2_,*D*_1,3_,*D*_2,1_,*D*_2,2_,*D*_2,3_,*D*_3,1_,*D*_3,2_,*D*_3,3_}. A target can be monitored only when it is within the sensing range of at least one directional sensor. Figure 1(a) shows that *R*_1_ = {*D*_3,3_}, *R*_2_ = {*D*_1,1_}, *R*_3_ = {*D*_2,3_}, *R*_4_ = {*D*_2,3_} and *R*_5_ = {*D*_1,1_}. From this figure, we can know that {*r*_2_,*r*_5_}, {*r*_3_,*r*_4_}, and {*r*_1_} are monitored simultaneously by *s*_1_, *s*_2_ and *s*_3_ (more specifically by *D*_1,1_, *D*_2,3_ and *D*_3,3_). Therefore, {*D*_1,1_,*D*_2,3_,*D*_3,3_} can represent a cover set. If two directions in *s*_1_ and *s*_3_, *D*_1,1_ and *D*_3,3_, are switched to *D*_1,2_ and *D*_3,1_, respectively, we can obtain a new cover set. Figure 1(b) shows an example for this case. From this figure, we can obtain a cover set {*D*_1,2_,*D*_2,3_,*D*_3,1_}.

The main objective of this paper is to maximize the network lifetime of a DSN. After all sensors are randomly scattered to monitor all the targets in a given target area, they have a fixed location. Without loss of generality, we can assume that all sensors have equal-number of directions and initially the same battery power. Then, the directions of sensors can belong to multiple cover sets, each of which has a different active time (*i.e.*, *t_k_*). If a cover set is determined to be active state by a target coverage scheduling mechanism, the directions in the cover set consume their sensor’ energy during the active time. The more cover sets we found, therefore, the longer the network lifetime we achieved. As a result, maximizing the network lifetime of DSNs is translated to how many cover sets can be found.

We organize the directions in *D* into *K* cover sets, where *K* is the maximum number of cover sets for a given coverage relationship between *S* and *R*. As *D_i,j_* can belong to multiple cover sets until the lifetime of a sensor *s_i_*, *L_i_*, completely runs down, we can define a boolean variable *x_i,j,k_* as in [15]:

(1) $$x_{i,j,k}=\{\begin{array}{ll} 1 & \text{if }D_{i,j}\in C_{k}\\ 0 & \text{otherwise}. \end{array}$$

We define the *MSCD (Maximum Set Covers for DSNs) problem* as follows.

(2) $$\text{Maximize}\;\;\;\sum_{k=1}^{K}t_{k}$$

(3) $$\text{subject}\;\text{to}\;\;\;\sum_{k=1}^{K}\sum_{j=1}^{W}x_{i,j,k}\cdot t_{k}\leq L_{i},\;\forall s_{i}\in S$$

(4) $$\sum_{j=1}^{W}x_{i,j,k}\leq 1,\;\forall s_{i}\in S,\;k=1,2,\ldots ,K$$

(5) $$\begin{array}{ll} & \sum_{D_{i,j}\in R_{m}}x_{i,j,k}\geq 1,\;\forall r_{m}\in R,\;k=1,2,\ldots ,K\\ \text{where} & x_{i,j,k}=\{0,1\}\;\text{and }t_{k}\geq 0 \end{array}$$

Equation (3) guarantees that the time allocated for each sensor *s_i_* across all cover sets is not larger than *L_i_*, which is the lifetime of each sensor. Equation (4) guarantees that one directional sensor in a cover set has at most one orientation depending on whether it is activated. Finally, Equation (5) guarantees that each target is covered by at least one direction in a cover set.

### 3.2. Greedy Algorithm

**Figure 2.**
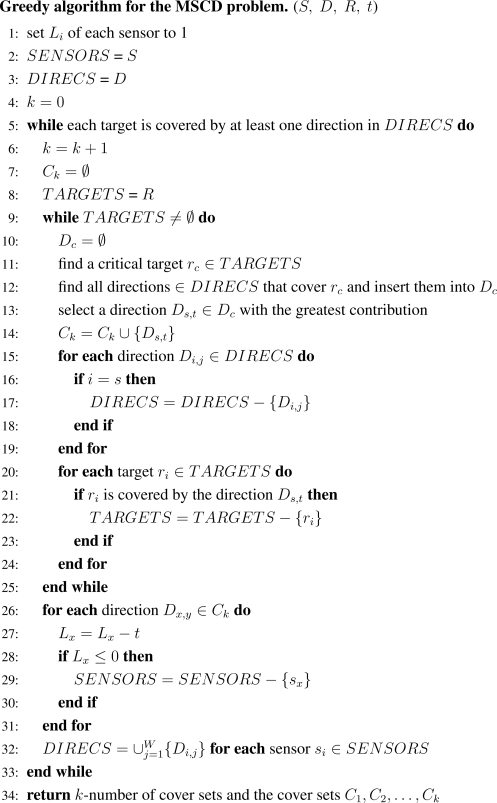
The greedy algorithm to solve the MSCD problem.

Figure 2 describes the details of the greedy algorithm devised to solve the MSCD problem using the same active time *t* for all cover sets. It is similar to one proposed previously [4], but it has been modified to capture the characteristics of DSNs. Our algorithm takes as input four parameters: *S* (the set of directional sensors), *D* (the set of directions), *R* (the set of targets), and *t* (the allocated active time of each cover set). This algorithm consists of the following steps:

**Step 1** Initialize the energy of each sensor and the variables *SENSORS*, *DIRECS*, and *k*. (lines 1–4).
**Step 2** Increase *k* by 1 and initialize the *k*th cover set and the variable *TARGETS* (lines 6–8).
**Step 3** Initialize the variable *D_c_* and a *critical target r_c_* is selected (lines 10–11). As the critical target, we select the target most sparsely covered in terms of the number of sensors.
**Step 4** Once the critical target *r_c_* has been selected, our algorithm selects the direction *D_s,t_* with the greatest contribution that covers the critical target (lines 12-13). Various contribution functions can be defined. In this paper, we use the following function *F* :
  (6) $$F(D_{i,j},r_{c})=\alpha \cdot N_{i,j,c}+(1-\alpha )\cdot L_{i},\;\;\;0\leq \alpha \leq 1.$$
  (7) $$D_{s,t}=\text{arg}\underset{D_{i,j}}{\text{max}}F(D_{i,j},r_{c}).$$
  where *N_i,j,c_* denotes the number of targets covered by the direction *D_i,j_* while *D_i,j_* already covers the target *r_c_*. By choosing an appropriate value of *α*, the direction *D_s,t_* will be selected such that it covers a larger number of uncovered targets and the sensor *s_s_* with the selected direction has more residual energy available.
**Step 5** Once a direction *D_s,t_* has been selected, it is added to the current cover set *C_k_* (line 14), and other directions of the same sensor *s_i_* are removed from the *DIRECS* set (lines 15–19).
**Step 6** All targets additionally covered by *D_s,t_* are removed from the *TARGETS* set (lines 20–24). When all targets are covered, a new cover set is formed. The condition in line 9 guarantees that a new cover set will cover all targets.
**Step 7** After a cover set *C_k_* has been formed, the lifetime of each sensor in *C_k_* is updated (lines 26–31). Once a sensor finishes its lifetime, it is removed from the set of available sensors, *SENSORS*.
**Step 8** Before going to line 5 to find a new cover set, the set of available directions *DIRECS* is updated based on the set *SENSORS* (line 32).

In Step 3, the concept of the critical target is used as a criterion of target selection. The critical target is defined as the target covered by least number of sensors. More importantly, it is a bottleneck in the view point of network lifetime; *i.e.*, when the energy of the sensors that cover the critical target is completely exhausted, the target cannot be covered anymore and hence the network lifetime is terminated. As a result, any cover set must contain the sensors monitoring the critical target. Our greedy algorithm first selects the critical target and then finds a direction of sensors that covers the target.

We use the same value of *t* for all cover sets during executing the greedy algorithm (and also during executing the genetic algorithm presented in the next section). But, it should be noted that all the cover sets found by one of both algorithms are not distinct. If some cover sets are found identical (e.g., *C_i_* = {*D*_1,1_,*D*_2,3_,*D*_3,2_} and *C_j_* = {*D*_1,1_, *D*_2,3_, *D*_3,2_}, for *i* ≠ *j*), they can be united, so that the united cover set {*D*_1,1_,*D*_2,3_,*D*_3,2_} is used for schedule and its active time is *τ* · *t* where *τ* is the number of the identical cover sets. It indicates that although we use the same value of *t* for all cover sets, each active time of actual cover sets will be different.

So far, we have described the greedy algorithm-based target coverage scheduling scheme to solve the MSCD problem. Even if this scheme can find the cover sets to maximize the network lifetime of a DSN in real time, its performance is extremely sensitive to how close an initial candidate is to an optimal solution. Thus, the scheme can lead to a local minimum solution due to its heuristic search. A more sophisticated method to solve the MSCD problem is required. In the next section, we will propose a genetic algorithm-based target coverage scheduling scheme that can find the optimal solution to the MSCD problem by evolutionary global search. In this paper, the greedy algorithm-based scheme will be used as a baseline for comparison.

## 4. Extending Network Lifetime by Genetic Algorithms

This section presents a genetic algorithm-based target coverage scheduling scheme that can solve the MSCD problem. After describing an overview of genetic algorithms, we present a detailed model of our genetic algorithm to find an optimal solution for extending the network lifetime of a DSN.

### 4.1. Overview of Genetic Algorithms

Genetic algorithms have attempted to mimic some of the processes taking place in natural evolution. Intuitively, they proceed by creating successive generations of better solutions by applying genetic operations [22]. The main application of genetic algorithms is optimization, where the goal is to find a set of parameter values that maximize performance on a given problem. The critical advantage of genetic algorithms is that genetic algorithms improve the chance of reaching the global optimum and also help in avoiding local optima [23].

The modeling of genetic algorithms for a given problem includes four basic steps: representation, fitness function, reproduction, and genetic operators. Representation is the encoding process converting the problem’s phenotypes into genotypes; *i.e.*, a candidate solution is represented by a chromosome and an element of the candidate solution is encoded into a gene. Each chromosome can be thought of as a point in the search space of candidate solutions. The performance of each chromosome is measured by the fitness function designed to be suitable for a given problem; *i.e.*, the fitness of a chromosome depends on how well that chromosome solves the problem. The evolution of genetic algorithms processes populations of chromosomes, successively replacing one population with another by reproduction. Genetic operators such as crossover, mutation, *etc.*, are used to generate a better chromosome in the next generation.

In the next subsections, we describe the detailed steps for modeling of a genetic algorithm to solve the MSCD problem.

### 4.2. Representation

**Figure 3.**
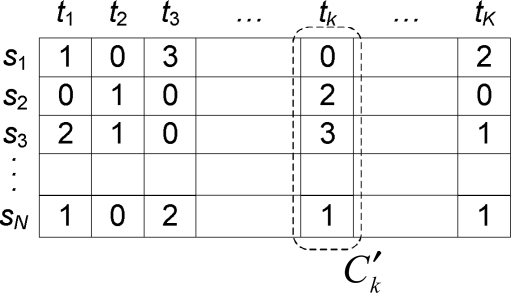
Chromosome representation.

Each chromosome in a population represents a candidate solution encoded as the direction of sensors for the MSCD problem. Figure 3 illustrates the two-dimensional chromosome represented as a grid with *N* rows and *K* columns. In Figure 3, a gene *g_i,k_*, which means the *i*th row and the *k*th column (*i* = 1,2,…,*N*, *k* = 1,2,…,*K*) in the chromosome, is interpreted as

(8) $$g_{i,k}=\{\begin{array}{ll} 0 & \text{if}\;\text{sensor}\;s_{i}\;\text{is}\;\text{sleep}\\ j & \text{if}\;D_{i,j}\;\text{of}\;\text{sensor}\;s_{i}\;\text{is}\;\text{active}\;\text{for}\;j=1,2,\ldots ,W. \end{array}$$

When an initial population is constructed, every gene in the chromosome is randomly set to an integer value using Equation (8). As described in [4], the value of *K* (the number of cover sets) is upperbounded by $\frac{d}{t}$, where *d* is the number of directions that cover the most sparsely covered target at an initial deployment, and *t* is the allocated active time of each cover set. This means that our genetic algorithm can find at most $\frac{d}{t}$ cover sets (*i.e.*, $K\leq \frac{d}{t}$). As more targets in an initial deployment are dependently covered by specific directions, the $\frac{d}{t}$ becomes smaller. As excessive chromosome size leads to the unsolicited search process in our genetic algorithm, we set a chromosome size to $\frac{d}{t}$ according to the initial deployment in a DSN.

One column in the chromosome shown in Figure 3 corresponds to a candidate cover set *C*′*_k_*. Such a candidate becomes a cover set *C_k_* when the following three conditions are all satisfied:

**Condition 1** All the targets should be covered by the directions in *C*′*_k_*.
**Condition 2** *C*′*_k_* should have directions each of which can cover at least one target.
**Condition 3** When target sets *T*_1_ and *T*_2_ are covered respectively by any two directions *D*_1_ and *D*_2_ in *C*′*_k_*, *T*_1_ ⊄ *T*_2_ and *T*_2_ ⊅ *T*_1_.

The total network lifetime is calculated as *K*′ · *t* (0 ≤ *K*′ ≤ *K*) by counting *C*′*_k_* satisfying the above conditions for *k* = 1,2,…,*K*.

### 4.3. Fitness Function

The two-dimensional chromosome presented in Figure 3 is evaluated to find efficient cover sets with a fitness function. To extend the network lifetime maximally, the evolutionary process of our genetic algorithm should achieve two sub-objectives: (1) the network lifetime should be extended with as many cover sets as possible and (2) the residual energy of each sensor should be maximized in the sense that new cover sets can be found by exhausting the residual energy until the network lifetime is not extended any longer. These two sub-objectives are made reasonable by the following considerations. When two chromosomes have an identical network lifetime, a chromosome with high residual energy will be beneficial to make the network lifetime more extended; *i.e.*, if the residual energy of each sensor is enough to construct one or more cover sets, the network lifetime can be eventually extended. Particularly, this will be more effective when the network lifetime is high. To achieve these two sub-objectives, we define a fitness function *f* as follows:

(9) $$o_{1}=\frac{K^{\prime }}{d/t},\;\;\;o_{2}=\text{tanh}\left(\kappa \cdot \sum_{i=1}^{N}L_{i}\right)$$

(10) $$f=\omega_{1}\cdot o_{1}+\omega_{2}\cdot o_{2}$$

where *o*_1_ and *o*_2_ represent sub-objective functions for the network lifetime and the residual energy of all sensors, respectively. *d* represents the number of directions that cover the most sparsely covered target at an initial deployment in a DSN. *κ* represents a slope parameter for the hyperbolic tangent function (*κ* *>* 0). *ω*_1_ and *ω*_2_ represent the parameters determining the significance of two sub-objective functions, respectively.

In Equation (9), *d/t* is used to scale the value of *o*_1_ the range from zero to one. For the sub-objective function *o*_2_, we use a hyperbolic tangent function due to its smoothness property. Using the slope parameter *κ*, we can easily adjust its shape. Since both *κ* and *L_i_* are positive, the value of *o*_2_ is also from zero to one. To make the evolutionary process more efficient, the values of *ω*_1_ and *ω*_2_ presented in Equation (10) are set according to the following relation: 0 ≤ *ω*_2_ *<* *ω*_1_ ≤ 1. This means that the first sub-objective function (*o*_1_) has much influence on achieving the maximization of the network lifetime than the second sub-objective function (*o*_2_). *o*_2_ is used as an auxiliary function to extend the network lifetime.

### 4.4. Reproduction

The reproduction process is the core of a genetic algorithm. In the reproduction process, selection mechanisms are used to organize a new population from the current population [22]. Among various selection mechanisms available, we use the following two ones.

- **Elitist:** The best runner and runner-up chromosomes are chosen in a current population and copied to a new population without any changes; *i.e.*, these two chromosomes survive into the next generation.
- **Roulette wheel:** The selection of chromosomes from the current population depends on the proportion of each chromosome’s fitness to the total fitness. As the fitness becomes higher, the probability that chromosomes will be chosen as parents in the current population increases; *i.e.*, chromosomes with high fitness are copied to the population of the next generation at a higher rate.

Given the population size *P*, *P* − 2 chromosomes in a new population are made by the roulette wheel selection mechanism. The elitist selection mechanism is applied to produce the remaining two chromosomes.

### 4.5. Genetic Operators

Here, we describe crossover and mutation operators to achieve the extended network lifetime in a DSN. In general, a crossover is a process that takes two parents and produces offstring from them with the aim of obtaining better chromosomes in the next generation. After a crossover point is randomly chosen, the part from the beginning of chromosome to the crossover point is copied from one parent, and the rest is copied from the second parent [22]. We design two types of crossover suitable for the structure of the two-dimensional chromosome shown in Figure 3.

**Figure 4.**
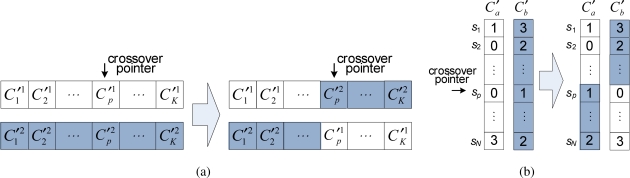
Example of two crossover operations. **(a)** Crossover for inter-cover sets; **(b)** Crossover for intra-cover sets.

- **Crossover for inter-cover sets:** This crossover is used for cover set exchange between two chromosomes; *i.e.*, cover sets in two chromosomes are exchanged with each other. Figure 4(a) shows an example of this crossover operation. By exchanging cover sets with each other in units of chromosomes, this crossover can produce chromosomes that include a larger number of cover sets, and therefore, we expect that the network lifetime will be extended.
- **Crossover for intra-cover sets:** This crossover is applied for two cover sets within a chromosome. Figure 4(b) illustrates an example of this crossover operation. Once two cover sets *C*′*_a_* and *C*′*_b_* (1 ≤ *a < b* ≤ *K*) are randomly chosen, directions in the cover sets are exchanged with each other to find a new cover set consisting of more energy-efficient directional sensors.

Mutation is used to maintain the genetic diversity in a population [22]. The mutation occurs at each gene in a cover set with a mutation probability *p_m_*. A gene *g_k,i_* for the direction of a sensor *s_i_* in a cover set *C*′*_k_* (1 ≤ *k* ≤ *K* and 1 ≤ *i* ≤ *N*) is changed to a new gene *g*′*_k,i_* as follows.

(11) $$g_{k,i}^{\prime }=\{\begin{array}{ll} j & \text{if}\;0\leq \beta <\frac{1}{3}\\ \left(g_{k,i}-1\right)\%(W+1) & \text{if}\;\frac{1}{3}\leq \beta <\frac{2}{3}\\ \left(g_{k,i}+1\right)\%(W+1) & \text{if}\;\frac{2}{3}\leq \beta \leq 1 \end{array}$$

where *j* is a random value ranging from 0 to *W*. In Equation (11), according to a random value *β*, a sensor *s_i_* can switch its current direction to the randomly chosen direction. It can also switch to the left or right direction from the current direction.

## 5. Performance Evaluation

In this section, we evaluate and analyze the performance of the proposed two schemes through simulations. The performance comparison for the schemes is also presented.

### 5.1. Simulation Environment

To conduct our simulations, we implemented a simulator with JDK 6.0. Using the simulator, we constructed a simulation environment to build a directional sensor network environment.

**Figure 5.**
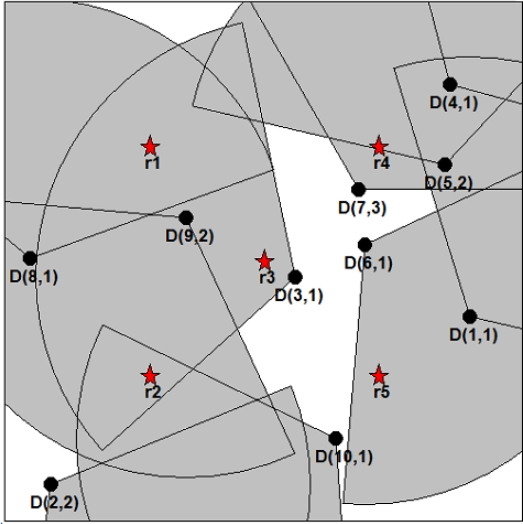
An example of target and sensor deployment.

Our simulation environment assumes that the different numbers of targets (*M* = 5 and 10) are uniformly deployed in a region of 500 m × 500 m and that the different numbers of directional sensors (*N* = 10, 20, 30, 40, and 50) are randomly scattered in the region. It also assumes that all the sensors can sense one of three directions, each of which has a direction angle of $\frac{2\pi }{3}(W=3)$ and no overlap with the other two directions. Various sensing ranges from 150 m to 300 m are used. Figure 5 shows an example of an initial target and sensor deployment in our simulation environment when five targets are covered by 10 directional sensors with one of three directions and a sensing range of 250 m.

In the greedy algorithm-based target coverage scheduling scheme, the parameter of a contribution function (*α* in Equation (6)) is set to 0.5. In the genetic algorithm-based scheme, chromosomes with $K\left(=\frac{d}{t}\right)$ cover sets are encoded to represent candidate solutions. Each gene in these chromosomes is initially set to a random value with the range of [0,3]. When simulations for these two schemes are conducted, the initial lifetime of all directional sensors (*L_i_* for *i* = 1,…,*N*) is set to 1.0, and the active time of all cover sets (*t*) is set to 0.1. Table 1 summarizes the parameters and values used in our simulations.

Our greedy algorithm-based and genetic algorithm-based target coverage scheduling schemes are evaluated according to the following three experimental factors.

- Number of directional sensors: This is used to investigate whether the two schemes solve the MSCD problem defined in our paper. We then compare the performance of the two schemes in terms of how much the network lifetime is extended with the different numbers of targets and directional sensors.
- Sensing ranges: This is used to investigate the performance of the two schemes with regard to the diverse sensing ranges of directional sensors. As the sensing ranges grow narrower, the target coverage of directional sensors shrinks. We expect that wider sensing ranges would lead to a larger number of cover sets than the narrower ranges.
- Distribution of directional sensors with different sensing ranges: When directional sensors have different sensing ranges, it is important to investigate the effects to find optimal cover sets. We will make the distribution of the number of directional sensors with different sensing ranges and then analyze how the distribution affects the performance of the two schemes.

In the next subsections, we present the simulation results to analyze the effect of these factors on the network lifetime and compare the performance of the two schemes in terms of the network lifetime of DSNs. The results presented here have been average over 10 simulation runs.

### 5.2. Effect of the Number of Directional Sensors

To investigate the influence of the number of directional sensors, we fixed the sensing ranges to 250 m. Directional sensors from 10 to 50 were used to cover 5 and 10 targets, respectively, and the performance was evaluated for each of the two schemes.

**Figure 6.**
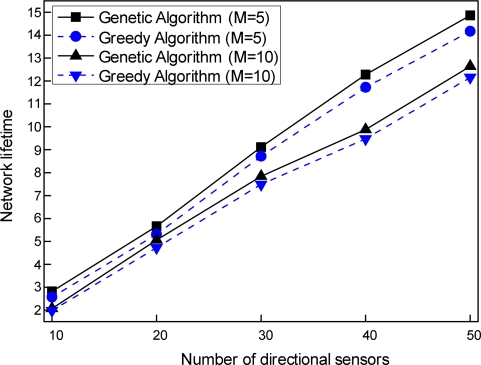
Comparison of network lifetimes according to the number of directional sensors.

Figure 6 shows the effect of the number of directional sensors on the network lifetime of the two schemes. As shown in this figure, the network lifetime for 5 targets is longer than that for 10 targets. This is because fewer targets would be covered by a smaller number of directions in cover sets, consuming less energy of directional sensors. For both of the schemes, therefore, as the number of targets to be covered is reduced, the network lifetime of each scheme increases. The results presented in this figure also indicate that the network lifetime increases almost linearly when the number of directional sensors increases. It should be noted that the increase in number of directional sensors can lead to finding more cover sets, as a greater number of directions for target coverage can be used for cover set construction.

Comparing the two schemes with regard to their network lifetime, our genetic algorithm-based target coverage scheduling scheme markedly extends the network lifetime compared with our greedy algorithm-based scheme, regardless of the number of directional sensors. This result indicates that the genetic algorithm-based scheme can find an optimal solution to the MSCD problem by global evolutionary search, in contrast to the greedy algorithm-based scheme, which is dependent on a heuristic search.

**Figure 7.**
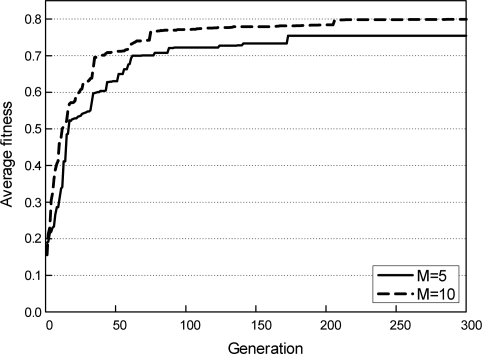
Evolution process of average fitness for ten runs of our genetic algorithm.

The evaluation process of our genetic algorithm-based scheme is shown in Figure 7, where the progress of average fitness for ten runs of our genetic algorithm is plotted. In Figure 7, a solid line represents the average fitness when 5 targets (*M* = 5) are covered by 10 directional sensors. The dotted line represents the average fitness when 10 targets (*M* = 10) are covered by 10 directional sensors. We can observe from this figure that two fitness curves grow higher as the generation increases. Therefore, we can see that our genetic algorithm-based scheme can sufficiently find the global optimum for the MSCD problem.

### 5.3. Effect of Sensing Ranges

In this simulation, we examined the performance of the two schemes according to the changes in sensing ranges of directional sensors. The lifetime variation was evaluated with the sensing ranges from 150 to 300 m when 10, 30, and 50 directional sensors were used to cover 5 and 10 targets, respectively.

**Figure 8.**
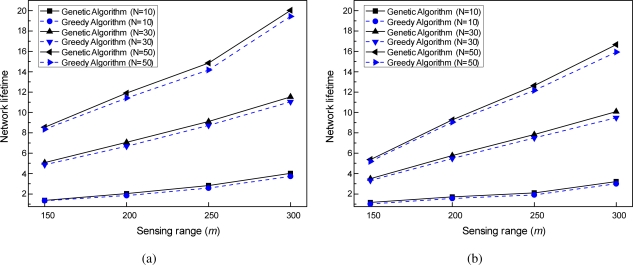
Comparison of network lifetimes according to changes in sensing ranges. **(a)** M = 5; **(b)** M = 10.

Figure 8 shows the effect of sensing ranges on the performance of the two schemes. The results presented in this figure indicate that the network lifetime becomes longer as the sensing ranges increase. This is not surprising; the wide sensing ranges can cover a greater number of targets than the narrow ranges, and thus fewer directions are used to construct cover sets. By finding as many such cover sets as possible, the overall network lifetime can be extended. This tendency can be observed regardless of the number of directional sensors and targets. Therefore, we can see that the wide sensing ranges cause the network lifetime to be extended in DSNs.

The results presented in Figure 8 also indicate that the genetic algorithm-based target coverage scheduling scheme can increase the network lifetime compared to the greedy algorithm-based scheme regardless of the sensing ranges of directional sensors. These observations indicate that, as compared with the greedy algorithm-based scheme, the genetic algorithm-based scheme can find a solution close to global optimum to MSCD problem without any influence of the sensing ranges used. Interestingly, when a sensing range of 150 m is used, the network lifetimes of the two schemes are almost indistinguishable visually. This is because the number of directions in an initial sensor and target deployment in itself is too small to obtain many cover sets. Nevertheless, the results shown in Figure 8 indicate that the genetic algorithm-based scheme can find at least as many cover sets as the greedy algorithm-based scheme. However, for the sensing ranges of more than 200 m, the network lifetime of the genetic algorithm-based scheme is consistently longer than that of the greedy algorithm-based scheme.

### 5.4. Effect of Distribution of Directional Sensors with Different Sensing Ranges

In this simulation, we evaluated the effect of the distribution of directional sensors with different sensing ranges on the performance of the two schemes. To evaluate this effect, we made a distribution of directional sensors with respect to sensing ranges. Table 2 shows the distribution of directional sensors classified into three types by three different sensing ranges, 200 m, 250 m, and 300 m. In this table, type *A* represents an identical number of directional sensors for the three sensing ranges used to cover targets in a region of 500 m × 500 m. In type *B*, the directional sensors with a sensing range of 300 m are much more common than those with the other two sensing ranges. This means that a DSN is mostly composed of the directional sensors with wide sensing ranges in an initial sensor deployment. Type *C* is the opposite of type *B*. Under this distribution, the network lifetimes are compared in accordance with the number of directional sensors (*N* = 10, 30, and 50) for 5 targets. The remaining parameters are the same as in the previous simulations.

**Figure 9.**
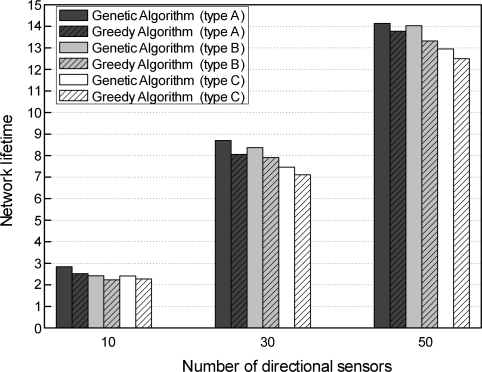
Comparison of network lifetimes for distribution of directional sensors with different sensing ranges.

Figure 9 shows the effect of the distribution of directional sensors with different sensing ranges on the performance of the two schemes. The network lifetime of the genetic algorithm-based scheme is longer than that of the greedy algorithm-based scheme, regardless of the types presented in Table 2. This indicates that the genetic algorithm-based scheme can extend the network lifetime significantly by global evolutionary search, as compared with the greedy algorithm-based scheme.

The network lifetime of type *A* is longer than that of the other two types (*B* and *C*). As the number of directional sensors increases, the number of directions for target coverage also increases. This means that our genetic algorithm-based scheme can find the cover sets evenly composed of directions with the three sensing ranges without having any influence on directions with a specific sensing range.

## 6. Conclusions

This paper discussed the target coverage scheduling for DSNs. In contrast to conventional sensor networks, DSNs are composed of a number of directional sensors with limited sensing ranges and directions, and thus target scheduling to maximize the network lifetime requires a highly sophisticated optimization technique. We have presented two target scheduling schemes, the greedy algorithm-based and the genetic algorithm-based schemes, to solve the MSCD problem that is known to be NP-complete. Throughout our simulations, different numbers of directional sensors, various sensing ranges, and heterogeneous directional sensors were used to investigate the effect of each on the performance of the two schemes. Simulation results showed that the schemes can find the cover sets monitoring all the targets in an energy-efficient way. They also showed that, by an evolutionary global search technique, the genetic algorithm-based scheme achieves a longer network lifetime than the greedy algorithm-based scheme does. As our future work, we plan to extend the schemes to maximize the network lifetime of DSNs considering the multi-hop connectivity of sensors as well as the coverage of the given targets.

## Figures and Tables

**Table 1.** Parameters and values used in our simulations.

Parameters	Values
Number of targets (*M*)	5, 10
Number of directional sensors (*N*)	10, 20, 30, 40, 50
Number of directions (*W*)	3
Sensing range	150 m, 200 m, 250 m, 300 m
Population size (*P*)	100
Number of generations	300
Crossover probability (*p_c_*)	0.1
Mutation probability (*p_m_*)	0.05
Slope parameter for *o*_2_ (*κ*)	0.3
Weighted parameter for *o*_1_ (*ω*_1_)	0.9
Weighted parameter for *o*_2_ (*ω*_2_)	0.1

**Table 2.** Distribution of directional sensors with different sensing ranges.

Type	Sensing ranges (m)		
	200	250	300
*A*	33%	33%	33%
*B*	20%	20%	60%
*C*	60%	20%	20%
